# Effects of emotion recognition training on mood among individuals with high levels of depressive symptoms: study protocol for a randomised controlled trial

**DOI:** 10.1186/1745-6215-14-161

**Published:** 2013-06-01

**Authors:** Sally Adams, Ian S Penton-Voak, Catherine J Harmer, Emily A Holmes, Marcus R Munafò

**Affiliations:** 1 School of Experimental Psychology, University of Bristol, 12a Priory Road, Bristol BS8 1TU, UK; 2 Department of Psychiatry, University of Oxford, Warneford Hospital, Warneford Lane, Oxford OX3 7JX, UK; 3 MRC Cognition and Brain Sciences Unit, 15 Chaucer Road, Cambridge CB2 7EF, UK

**Keywords:** Emotion recognition training, Mood, Depression

## Abstract

**Background:**

We have developed a new paradigm that targets the recognition of facial expression of emotions. Here we report the protocol of a randomised controlled trial of the effects of emotion recognition training on mood in a sample of individuals with depressive symptoms over a 6-week follow-up period.

**Methods/Design:**

We will recruit 190 adults from the general population who report high levels of depressive symptoms (defined as a score ≥ 14 on the Beck Depression Inventory-II). Participants will attend a screening session and will be randomised to intervention or control procedures, repeated five times over consecutive days (Monday to Friday). A follow-up session will take place at end-of -treatment, 2-weeks and 6-weeks after training. Our primary study outcome will be depressive symptoms, Beck Depression Inventory- II (rated over the past two weeks). Our secondary outcomes are: depressive symptoms, Hamilton Rating Scale for Depression; anxiety symptoms, Beck Anxiety Inventory (rated over the past month); positive affect, Positive and Negative Affect Schedule (rated as ‘how you feel right now’); negative affect, Positive and Negative Affect Schedule (rated as ‘how you feel right now’); emotion sensitivity, Emotion Recognition Task (test phase); approach motivation and persistence, the Fishing Game; and depressive interpretation bias, Scrambled Sentences Test.

**Discussion:**

This study is of a novel cognitive bias modification technique that targets biases in emotional processing characteristic of depression, and can be delivered automatically via computer, Internet or Smartphone. It therefore has potential to be a valuable cost-effective adjunctive treatment for depression which may be used together with more traditional psychotherapy, cognitive-behavioural therapy and pharmacotherapy.

**Trial registration:**

Current Controlled Trials: ISRCTN17767674

## Background

Mood disorders are highly prevalent in the general population, dominated by major depression, and constitute a substantial burden of disease. At present, National Institute for Clinical Excellence guidelines recommend psychotherapy for mild depression, and cognitive-behavioural therapy (CBT) for moderate depression [1], but these therapies require individual intervention and therefore, while cost-effective, are expensive. Here we propose a study to investigate the potential of a novel cognitive bias modification technique for reducing negative mood and improving positive mood in an analogue population of those with high levels of depression symptoms. This targets biases in emotional processing characteristic of depression, and can be delivered automatically via computer, Internet or Smartphone. Computerised programs for CBT have already been shown to improve accessibility to treatment and reduce symptoms of depression [2]. Their aim is to deliver existing talking-therapy CBT techniques via computer. An alternative emerging option is to use the computer to deliver novel techniques, which like CBT aim to alter biasing in thinking styles but do so in a rather different manner.

**Figure 1:**
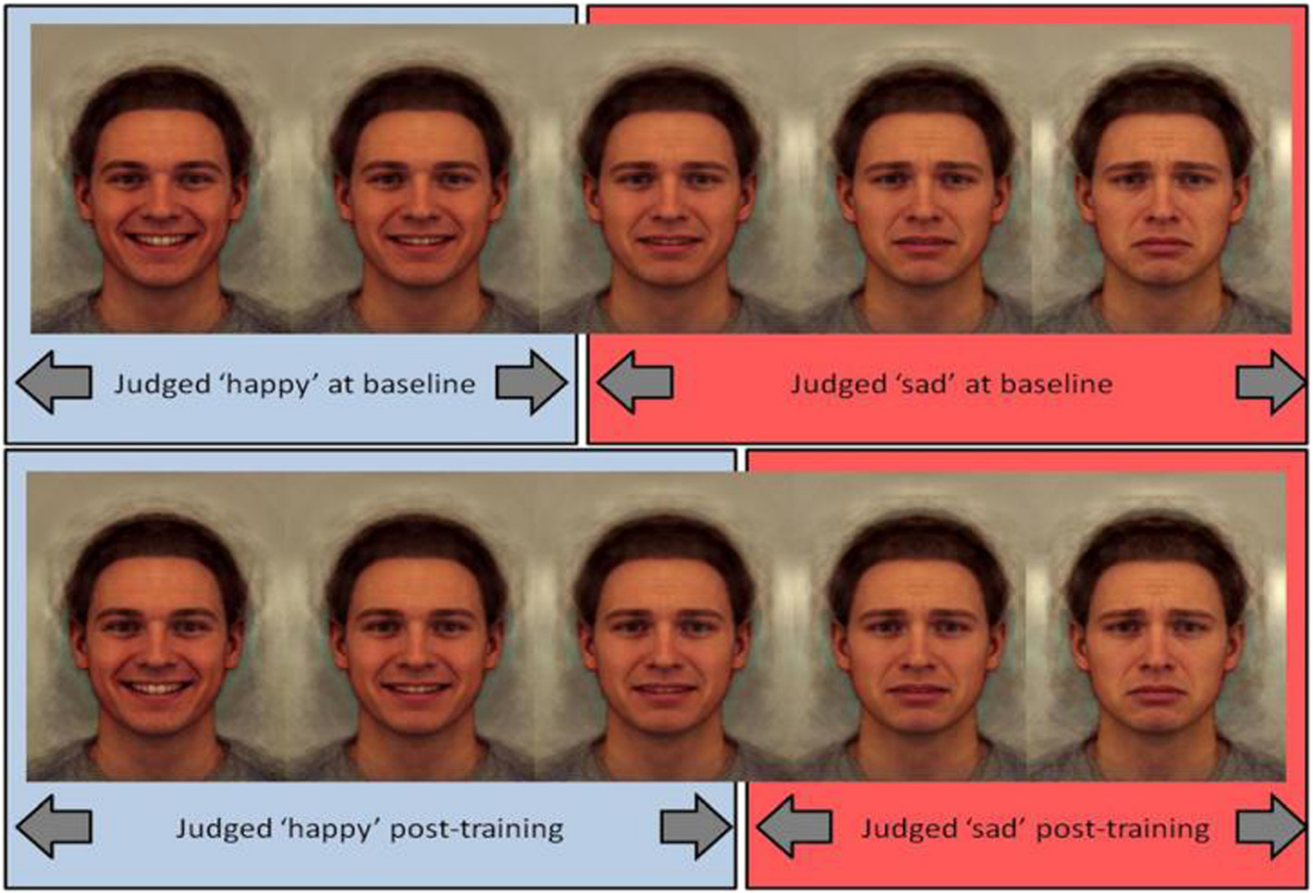
Sample of face morph sequence.

Cognitive-bias modification (CBM) encompasses a range of techniques designed to manipulate biases in cognitive processing characteristic of specific psychopathologies and involved in the maintenance of the disorder [3]. For example, attentional bias towards threat is a feature of anxiety disorders, and can be reduced through retraining procedures [4]. There have been innovations in CBM for a range of biases, including attention, interpretation, and memory. However, a key feature of emotion processing highly relevant to psychopathology has received little attention to date – the recognition of facial expression of emotions. Faces play a key role in everyday life, and the accurate recognition of emotional content in faces is critical to social functioning. This is disrupted in a range of psychiatric disorders – for example, people with depression show a negative bias whereby they fail to identify happiness in faces [5,6]. In particular, the negative bias characteristic of depression also predicts relapse in currently depressed and remitted patients [7]. This strongly suggests that such biases play a causal role in the aetiology and maintenance of depression. Indeed, recent advances in the CBM of depression have focused on promoting positive biases rather than merely reducing negative biases [8,9]. We have developed a new paradigm that targets the recognition of facial expression of emotions by initially assessing the threshold for detecting one emotion over another in an ambiguous expression (for example, a blend of happiness and sadness), and then providing feedback to shift this threshold (for example, to favour identification of happiness over sadness). Training consists of feedback to shift the participant’s balance point, estimated by presenting exemplar faces from a 15-frame morphed face image continuum using a two-alternative forced choice procedure. In the training condition, the ‘correct’ classification shifted two morph steps towards ‘happy’; the two images nearest the balance point that the participant would have previously classified as ‘sad’ at baseline were considered ‘happy’ in terms of providing feedback. Feedback in the control condition was based directly on baseline performance (Figure 1).

Preliminary results from adults recruited from the general population on the basis of high levels of depressive symptoms on the Beck Depression Inventory-II (BDI-II) show that this manipulation of the perception of emotion in ambiguous facial expressions, designed to promote the perception of positive emotion over negative emotion, may have therapeutic benefit that persists for at least two weeks [10]. The strength of the observed effect of training on positive mood appears to strengthen over time. This is consistent with recent models of the action of antidepressant medication, which suggest that drug treatment has early effects on emotional processing bias including the ability to detect positive versus negative facial expressions [11]. This is argued to result in therapeutic benefit (i.e., improved mood) only after sufficient time has elapsed to allow interaction with others, where alteration in these processing biases gives rise to more positive social interactions. Our intervention aims to target these biases directly through automated behavioural feedback. Here we propose taking the next critical step to translate this work to establish the therapeutic potential of this novel approach.

### Study objectives and hypotheses

The present project aims to establish the effects of emotion recognition training on mood in a sample of individuals with depressive symptoms over a 6-week follow-up period. Preliminary evidence suggests a beneficial effect of emotion recognition training on positive mood at 2-week follow-up following four consecutive days of training [10]. However, we would hypothesise that these effects would strengthen over time, by modifying the nature of social interaction through the perception of positive emotion over negative emotion. We will therefore investigate the longer-term effects of training over time, using a 6-week follow-up period. We hypothesise that individuals randomised to receive an intervention designed to modify emotion perception designed to increase the perception of happiness over sadness in ambiguous expressions will show reduced depressive symptoms, reduced negative affect, and increased positive effect, compared with individuals randomised to receive a placebo intervention.

## Methods/design

### Design

This study will examine the effects of emotion recognition training on a sample of individuals with depressive symptoms over a 6-week follow-up period. Participants will attend a screening session and will be randomised to intervention or control procedures, repeated five times over consecutive days (Monday to Friday). A follow-up session will take place at end-of-treatment, 2-weeks and 6-weeks after training.

### Participants and recruitment

We will recruit 190 adults from the general population who report depressive symptoms (defined as a score ≥ 14 on the BDI-II [12]). A score ≥ 14 is the standard cutoff value used to indicate mild depression, as described in the BDI-II scoring manual.

**Figure 2:**
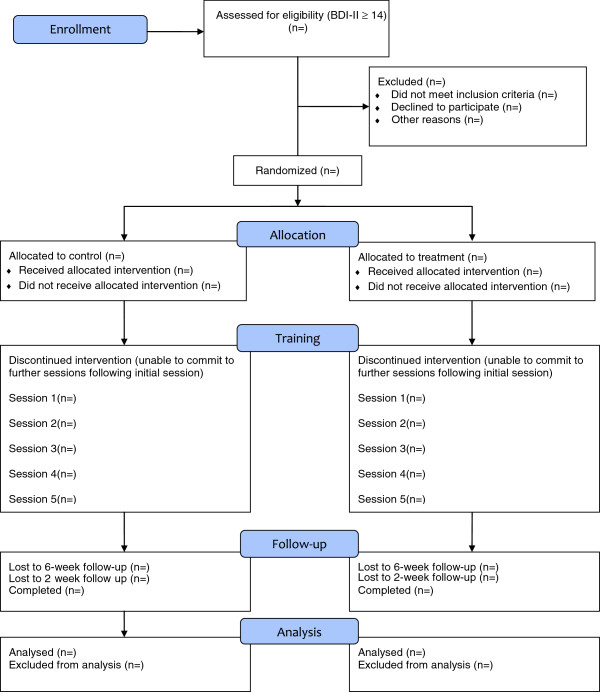
CONSORT flow diagram.

A CONSORT diagram of participant recruitment is shown in Figure 2. Participants will be recruited from the staff and students at the University of Bristol and the general population. Participants will be recruited by existing email lists, by poster and flyer advertisements, and by word of mouth. They will be asked to contact the research coordinator for further details of the study if they are interested in taking part. Those who meet the study inclusion criteria will be sent the information sheet, and asked to contact the research coordinator again if they would like to sign up for the study or if they require further information. On completion of the study session, participants will be reimbursed £60 for their time and expenses.

### Inclusion criteria

For inclusion, participants should: be aged between 18 and 40 years; score ≥ 14 on the BDI-II; have English as their first language or an equivalent level of fluency; and be able to give informed consent as judged by the lead researcher.

### Exclusion criteria

Criteria for exclusion are as follows: primary anxiety disorder, psychosis, bipolar disorder, or substance dependence (other than nicotine and caffeine) as defined by the Diagnostic and Statistical Manual of Mental Disorders, fourth edition; current use of an illicit drug (except cannabis); being at clinically significant risk for suicidal behaviour; use of psychotropic medication in the last 5 weeks prior to the study; major somatic or neurological disorders and concurrent medication that could alter emotional processing (including active treatment with counselling, cognitive behavioural therapy, or other psychotherapies) – we will allow intermittent use of medication, judged by the principal investigator; and participants who in the opinion of the lead researcher are not appropriate for participation. Participants who develop or engage in any of these criteria during the course of the study will be removed from the final analysis.

### Ethical considerations and informed consent

Ethics approval from the Faculty of Science Ethics Committee has been granted. The study will be conducted according to the revised Declaration of Helsinki and Good Clinical Practice guidelines. The investigator or co-investigator will explain the nature, purpose, and risks of the study to the participant. The participant will receive the information sheet. There will be no time restriction on how long participants take to respond, with the exception that participants who respond after all study places have been filled will not be offered a place on the study. Therefore, participants will be given sufficient time to read the information and consider any implications, and to raise any questions with the investigators prior to making a decision to participate. Participants will be informed that they are free to withdraw at any time.

### Sample size determination

Our preliminary data indicate an effect size of *d* = 0.43 at 2-week follow-up, corresponding to a difference of 3 points on the Positive and Negative Affect Schedule (PANAS). This indicates that a sample size of *n* = 172 will be required to achieve 80% power at an alpha level of 5%. This effect size may be conservative as our data indicate a trend for the effect of training to strengthen over time, which is consistent with our underlying theoretical model. This sample size will give us equivalent power to detect a difference of 5 points on the BDI-II. We will therefore recruit *n* = 190 participants, including 10% over-recruitment to accommodate potential attrition.

### Randomisation

Participants will be randomised to intervention or control procedures, repeated five times over consecutive days (Monday to Friday). Equal numbers of participants per group will be maintained (i.e., 95 intervention and 95 control). In advance of the study, an experimental collaborator (who will have no direct contact with the study participants) will prepare a numeric code using random number assignment software. Once a participant has passed the screening assessments, the lead researcher will contact the experimental collaborator who will provide treatment group allocation.

The lead researcher will enter the treatment group code into the computer and will not be present in the room while the participant completes the computer procedures to ensure that blinding is maintained.

### Study design

This study will examine the effects of emotion recognition training on a sample of individuals with depressive symptoms over a 6-week follow-up period. Participants will attend a screening session and will be randomised to intervention or control procedures, repeated five times over consecutive days (Monday to Friday). A follow-up session will take place at end-of-treatment, 2-weeks and 6-weeks after training.

### Primary outcome measures

The primary outcome measure is depressive symptoms, using the BDI-II (rated over the past 2 weeks).

### Secondary outcome measures

The secondary outcome measures are: depressive symptoms, using the Hamilton Rating Scale for Depression; anxiety symptoms, using the Beck Anxiety Inventory (rated over the past month); positive affect, using the PANAS (rated as ‘how you feel right now’); negative affect, using the PANAS (rated as ‘how you feel right now’); emotion sensitivity, using the Emotion Recognition Task (test phase); approach motivation and persistence, using the Fishing Game; and depressive interpretation bias, using the Scrambled Sentences Test.

### Procedures

We will recruit 190 adults from the general population who report depressive symptoms (defined as a score ≥ 14 on the BDI-II). Approximate timings of the screening day procedures are presented in Table 1. Participants will provide informed consent and inclusion/exclusion criteria will be assessed. Screening will consist of structured clinical interview for Diagnostic and Statistical Manual of Mental Disorders, fourth edition, using the Clinical Interview Schedule [13], the Altman Self-Rating Mania Scale (for bipolar disorder) [14], and medical history. After initial screening we will collect baseline data on age, sex, ethnicity, alcohol, tobacco and caffeine use, previous history of depression (treated and non-treated), intelligence (National Adult Reading Test) [15], number of years of education, social network size, and current and past history of psychiatric treatment. At screening we also will assess whether participants meet criteria for clinical depression, number of episodes of depression, age at first episode, and whether participants have ever taken antidepressant medication (including details of how recently).

**Table 1:** Timings of procedures of screening session (day 1)

**Time (minutes post arrival)**	**Task**
0	Informed consent
15	Screening
40	Baseline measures
50	Mood assessments
60	Computerised training
90	Depart

Participants will be randomised to intervention; a training procedure designed to promote the perception of happiness over sadness in ambiguous emotional expressions, or a control procedure designed to elicit no change in perception of emotional expression (described below). Participants will complete computerised training (or control) procedures repeated five times over consecutive days (Monday to Friday). Each session will consist of three phases: baseline, training, and test. Approximate timings of the treatment-day procedures are presented in Table 2.

**Table 2:** Timings of procedures of treatment sessions (days 2 to 5)

**Time (minutes post arrival)**	**Task**
0	Computerised training
20	Depart

Mood assessments (BDI-II, Beck Anxiety Inventory, Hamilton Rating Scale for Depression and PANAS) will be taken at baseline, at the end of treatment, and at 2-week and 6-week follow-up. Behavioural assessments (Emotion Recognition Task, Scrambled Sentences Test, and the Fishing Game) will be taken at the end of treatment, and at 2-week and 6-week follow-up. 6-week follow-up will also include a visual analogue scale rating of how helpful the participant thought the experimenter was, to ensure that there are no differences between treatment groups which may affect blinding. At 6-week follow-up will we reassess the initial exclusion criteria, to establish any change in these (Table 3).

**Table 3:** Timings of procedures of follow-up sessions (end of treatment, 2 weeks and 6 weeks)

**Time (minutes post arrival)**	**Task**
0	Mood assessments and behavioural tasks
20	Exclusion criteria (6-week only)
30	Visual analogue scale rating of experimenter (6-week only)
40	Depart

### Measures and materials

#### Training task

The baseline and test phases each consist of 45 trials, in which each of the stimuli from the morph sequence is presented to the participant three times. The task requires the participant to make a forced choice judgement as to whether the presented face is displaying a sad or happy expression. Images are presented one at a time, in random order, for 150 milliseconds. Stimuli are preceded by a fixation cross, which is presented for a random period ranging from 1500 to 2500 milliseconds. Subsequent to presentation, and to prevent processing of afterimages, a backward mask of noise is presented for 250 milliseconds, followed by a prompt asking the participant to respond. This remains on screen until the participant makes response (i.e., a judgement of ‘sad or ‘happy).

Each trial in the training phase is similar to a trial in the baseline and test phases with respect to inter-trial interval and stimuli presentation, but with the addition of feedback subsequent to the participant’s response. In the control condition, feedback is based on the participant’s baseline balance point. That is, responses are classified as ‘correct’ if the participant identified images below the original balance point image as ‘sad’ and above it as ‘happy’, and otherwise are classified as ‘incorrect’. Feedback is a message saying ‘Correct / Incorrect! that face was sad/happy’. In the training condition, feedback is again based on the participant’s baseline balance point, but the ‘correct’ classification is shifted two morph steps towards the ‘sad’ end of the continuum, so that the two images nearest the balance point that the participant would previously have classified as ‘sad’ at baseline are considered ‘happy’ when providing feedback. In each training block, each face from the 15 face continuum is presented twice. The order of presentation is randomised within each block. Six training blocks are given to each participant, resulting in 180 training trials in total.

Prototypical ‘sad’ and ‘happy’ composite images have been generated from 20 individual male faces showing a sad facial expression, and the same 20 individuals showing a happy expression. Original images come from the Karolinska directed emotional face set [16]. To construct these images, the 40 original images were each delineated with 172 feature points, which allows both shape and colour information to be averaged across the faces to generate ‘average’ sad and happy expressions, using established techniques [17]. These prototypical images were used as endpoints to generate a linear morph sequence that consists of images that change incrementally from unambiguously ‘sad’ to unambiguously ‘happy’, with emotionally ambiguous images in the middle. We have created a sequence with 15 equally spaced images for use as experimental stimuli (see Figure 1 for endpoints and example intermediate stimuli).

#### Approach motivation and persistence: the fishing Game

In this task, 12 brightly coloured plastic fish move round in a circle, opening and closing their mouths to reveal a magnet [18]. Participants are required to catch as many fish as they can in 2.5 minutes by ‘hooking’ them using a magnet on the end of a 900 mm plastic fishing rod. The fishing game task is a simple behavioural performance measure assumed to tap behaviour negatively associated with dysphoria, such as approach motivation and persistence.

#### Depressive interpretation bias: scrambled sentences test

In this task, participants unscramble a list of 20 scrambled sentences (e.g., ‘winner born I am loser a’) under a cognitive load (remembering a six-digit number) [19]. This task measures the tendency of participants to interpret ambiguous information either positively (‘I am a born winner’) or negatively (‘I am a born loser’). A negativity score is generated by calculating the proportion of sentences completed correctly with a negative emotional valence.

#### Social network size: friendship and social network size

In this task, participants rate the number of close friends they currently have on a 5-point scale ranging from 0 (none) to 4 (four or more) [20]. A close friend is defined as a person whom respondents report feeling close to and whom they believe they could confide in and turn to for help. Participants will also repeat this process, rating the number of contacts and acquaintances they currently have. A contact or acquaintance is defined as a person known by sight or known to someone, but not intimately.

### Statistical plan

We will use linear regression to evaluate the effect of training on mood at 6-week follow-up. Analyses will be adjusted for age, sex, ethnicity, previous history of treatment for depression, and baseline mood. The primary outcome will be depressive symptoms assessed using the BDI-II. Secondary outcomes will include depressive symptoms measured using the Hamilton Rating Scale for Depression, and positive and negative affect assessed using the PANAS.

Subgroup analyses by whether participants meet criteria for clinical depression, number of episodes of depression, age at first episode, and whether participants have depression with or without anxiety will also be conducted. We will also analyse the impact of social network size on treatment effect. We predict that larger social network will be associated with an enhanced treatment effect.

## Discussion

Here we propose a study to investigate the potential of a novel cognitive bias modification technique for reducing negative mood and improving positive mood in an analogue population of those with high levels of depression symptoms. This targets biases in emotional processing characteristic of depression, and can be delivered automatically via computer, Internet or Smartphone. This technique therefore has potential to be a valuable cost-effective adjunctive treatment for depression which may be used together with more traditional psychotherapy, CBT and pharmacotherapy.

Our approach is to model treatment development in analogue populations before development in the disorder of interest. In this case our analogue groups are people suffering from depressive symptoms (dysphoria) but are not selected on the basis of a clinical diagnosis. This approach allows us to refine the ingredients of successful intervention and to establish the best clinical study to subsequently carry out in a depressed cohort. In particular, it avoids potential confounding effects of medication, and allows fast recruitment of a group characterised by negative emotion processing biases. The approach is a well-validated method in the wider basic psychology literature, and provides a useful model of depression to explore treatment effects before translation into a full clinical trial.

## Trial status

This article was submitted on 19 September 2012. To date, one participant has been recruited. The first participant was enrolled on 3 September 2012.

## Abbreviations

BDI-II: Beck depression inventory II; CBT: Cognitive-behavioural therapy; PANAS: Positive and negative affect schedule.

## Competing interests

MM and IP-V are co-directors of Jericoe Ltd, which produces software for the assessment and modification of emotion perception.

## Authors’ contributions

MM, IPV, CH and EH participated in the conception and design of the trial and in plans for the analysis of the data. SA, MM, IPV, CH and EH drafted the manuscript. SA participated in data collection, and was responsible for recruitment and treatment of participants. All the authors discussed, read, revised the manuscript, and all approved the publication of this protocol.
